# 5-Bromo-4′,5′-bis(dimethylamino)fluorescein: Synthesis and Photophysical Studies

**DOI:** 10.3390/molecules23010219

**Published:** 2018-01-20

**Authors:** Jun Yeon Hwang, Jung-Yean Lee, Chang-Woo Cho, Wonjun Choi, Yejin Lee, Sangdeok Shim, Gil Tae Hwang

**Affiliations:** 1 Department of Chemistry and Green-Nano Materials Research Center, Kyungpook National University, Daegu 41566, Korea; gghog@naver.com (J.Y.H.); leejjung0102@gmail.com (J.-Y.L.); cwcho@knu.ac.kr (C.-W.C.); 2 Department of Chemistry, Sunchon National University, 255 Jungang-ro, Sunchon, Jeonnam 57922, Korea; chl5288018@naver.com (W.C.); leeyejin710@gmail.com (Y.L.)

**Keywords:** fluorescein, fluorophore, fluorescent probes, pH-sensitive probes, Suzuki coupling

## Abstract

In this study, three new fluorescein derivatives—5-bromo-4′,5′-dinitrofluorescein (**BDNF**), 5-bromo-4′,5′-diaminofluorescein (**BDAF**), and 5-bromo-4′,5′-bis(dimethylamino)fluorescein (**BBDMAF**)—were synthesized and their pH-dependent protolytic equilibria were investigated. In particular, **BBDMAF** exhibited pH-dependent fluorescence, showing strong emission only at pH 3–6. **BBDMAF** bears a bromine moiety and thus, can be used in various cross-coupling reactions to prepare derivatives and take advantage of its unique emission properties. To confirm this, the Suzuki and Sonogashira reactions of **BBDMAF** with phenylboronic acid and phenylacetylene, respectively, were performed, and the desired products were successfully obtained.

## 1. Introduction

Fluorescein is one of the most widely used fluorophores in the chemical, biochemical, and medicinal fields due to its ease of synthesis and brilliant green fluorescence [1,2,3,4,5,6]. Fluorescein shows complex protolytic equilibria in aqueous solution. The dianionic and monoanionic forms, which are present at pH > 6, exhibit bright fluorescence; however, the fluorescence of the neutral and monocationic forms, which are present at pH < 6, is drastically reduced. Therefore, great care must be taken when using fluorescein as a fluorescent probe under acidic or pH-ambiguous conditions. To overcome this disadvantage, studies have been carried out to lower the p*K*_a_ by attaching an electron-attracting group such as a halogen to the xanthene ring of fluorescein [7,8]. Nevertheless, these fluorescein derivatives, such as fluorescein, present the same disadvantage in that their emission decreases upon decreasing the pH.

**Figure 1:**
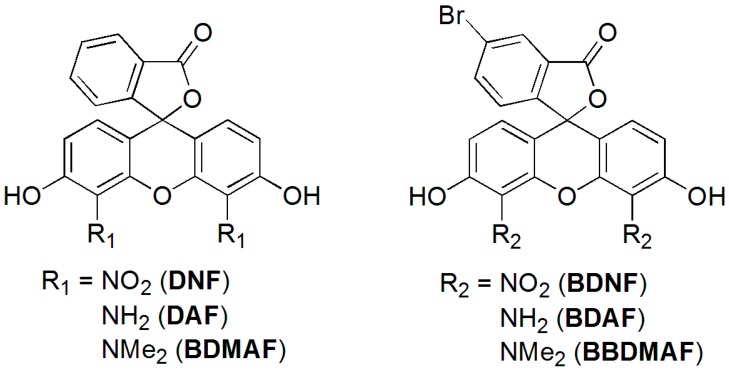
Fluorescein derivatives used in this study.

We recently reported 4′,5′-bis(dimethylamino)fluorescein (**BDMAF**)-bearing dimethylamino groups at the 4′ and 5′ positions of the xanthene ring (Figure 1) [9]. The most prominent feature of this fluorescein derivative is that, between pH 3 and 6, the photoinduced electron transfer (PET) process is inhibited by the protonation of the unpaired electrons of the nitrogen atoms resulting in an increased fluorescence in this pH region. In order to extend the applicability of **BDMAF**, we designed 5-bromo-4′,5′-bis(dimethylamino)fluorescein (**BBDMAF**) containing a bromine moiety at the 5 position of the benzoic acid. Since fluorescein derivatives bearing a bromine moiety can be used in various cross-coupling reactions [10,11,12], **BDMAF** is expected to be a key compound with applications in sensors and biomarkers. Here, we report the synthesis of **BBDMAF**, its protolytic equilibria, and its pH-dependent fluorescence.

## 2. Results and Discussion

### 2.1. Synthesis of **BDNF**, **BDAF**, and **BBDMAF**

**Scheme 1:**
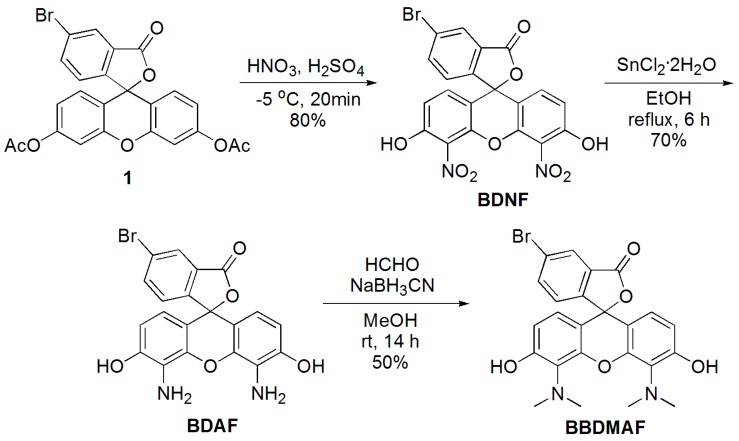
Synthesis of **BDNF**, **BDAF**, and **BBDMAF**.

Nitration of 5-bromofluorescein diacetate **1**, which was synthesized following the method reported by the Burgess group [13], afforded 5-bromo-4′,5′-dinitrofluorescein (**BDNF**). Reduction of **BDNF** by catalytic hydrogenation with palladium on carbon yielded 5-bromo-4′,5′-diaminofluorescein (**BDAF**); subsequent reductive amination with formaldehyde gave **BBDMAF** (Scheme 1).

### 2.2. pK_a_ Determination of **BDNF**, **BDAF**, and **BBDMAF**

**Figure 2:**
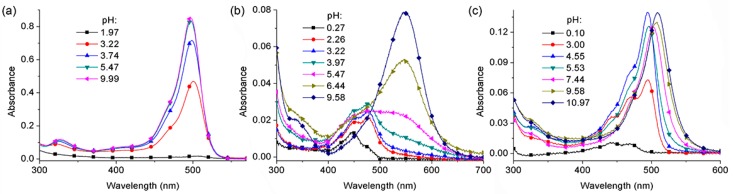
Selected absorption spectra of (**a**) **BDNF** (12.5 μM) in EtOH:10 mM phosphate buffer (1:1, *v*/*v*), (**b**) **BDAF** (2.5 μM), and (**c**) **BBDMAF** (2.5 μM) in 10 mM phosphate buffer at several pH levels at 25 °C. The spectra at pH 0.15–1.45 were obtained in HCl solution. All samples contain 0.05% DMSO to ensure solubility.

**Figure 3:**
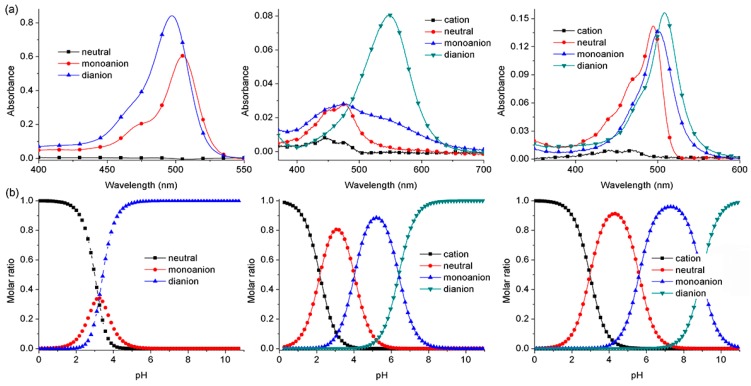
(**a**) Proposed spectra for the protolytic forms (cation, neutral, monoanion, and dianion) of **BDNF** (left), **BDAF** (middle), and **BBDMAF** (right) generated by the DATAN program; (**b**) relative concentration curves for each protolytic species versus pH.

The ultraviolet–visible (UV‒Vis) absorption spectra of **BDNF** (12.5 μM), **BDAF** (2.5 μM), and **BBDMAF** (2.5 μM) were recorded at various pH levels in mixtures of EtOH and 10 mM phosphate buffer (1:1, *v*/*v*) or in 10 mM phosphate buffer (Figure 2 and Figure S1). Relatively high amounts of **BDNF** compared to **BDAF** and **BBDMAF** were dissolved in 50% EtOH to dissociate the absorption bands of the monoanionic and dianionic forms, as previously reported for **DNF** [14]. The UV‒Vis absorption spectra and pH-dependent mole fractions of each protolytic species of **BDNF**, **BDAF**, and **BBDMAF** were predicted and their p*K*_a_ values were determined through the DATAN program analysis (Figure 3 and Table 1) [15,16,17].

**Scheme 2:**
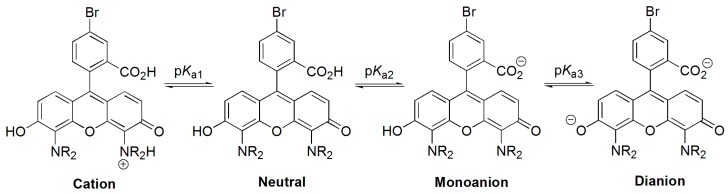
Possible protolytic equilibria of **BDAF** (R=H) and **BBDMAF** (R=CH_3_).

Similar to **DNF**, which does not contain a bromine moiety, **BDNF** displayed very low absorbance in the neutral form, and the absorption bands of the monoanionic and dianionic forms were very close to each other (Table S1). There was no significant difference in the p*K*_a1_ and p*K*_a2_ values of **BDNF**, as observed with **DNF** [14]. The values of p*K*_a1_ and p*K*_a2_, which relate to the protonation of the benzoate and phenolate forms of **BDNF**, were 3.19 and 3.22, respectively. This result indicated that the introduction of a bromine moiety into **BDNF** causes an increase in the acidity over that of **DNF**. **BDAF** and **BBDMAF** also displayed absorption bands similar to those of **DAF** and **BDMAF**, respectively [9]. Comparing the p*K*_a_ values of **BDAF** and **BBDMAF** with those of **DAF** and **BDMAF**, it was found that the introduction of a bromine moiety slightly reduced the p*K*_a1_ and p*K*_a2_ values, which relate to the protonation of the amino group and the benzoate, respectively. On the other hand, the value of p*K*_a3_, which represents the protonation of the phenolate, slightly increased. Based on these data, we suggest the protolytic equilibria of **BDAF** and **BBDMAF**, as shown in Scheme 2.

### 2.3. Fluorescence of **BDNF**, **BDAF**, and **BBDMAF**

**Figure 4:**
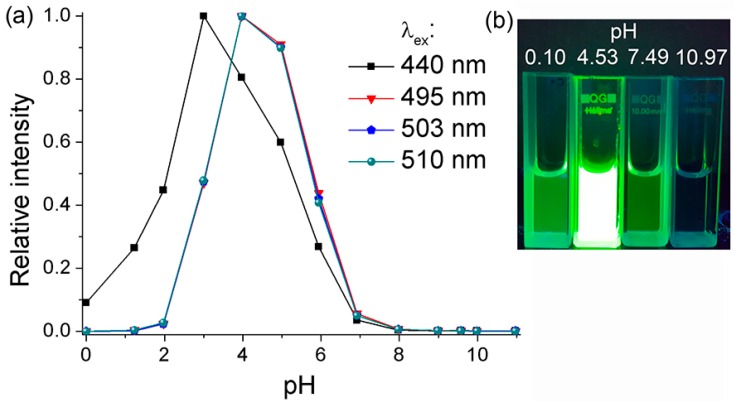
(**a**) Relative intensity of maximum emission of **BBDMAF** (515 nm) versus pH; each protolytic species was excited at its maximum absorption wavelength. (**b**) Emission image of **BBDMAF** solutions (concentration of each: 2.5 μm) illuminated under a UV transilluminator (312 + 365 nm).

The pH-dependent emission behaviors of **BDNF**, **BDAF**, and **BBDMAF** were next examined. Excitation was performed at the maximum absorption wavelength of the protolytic species of each dye at pH 0.00, 1.23, 1.97, 3.00, 3.97, 4.97, 5.94, 6.92, 7.98, 8.99, 9.58, 9.99, and 10.97 (Figure S2). The fluorescence of **BDNF** and **BDAF** was almost quenched, regardless of the pH and excitation wavelength. However, **BBDMAF** was found to show a similar pH-dependent fluorescence behavior as **BDMAF**. Excitation at the maximum absorption wavelength of the cationic **BBDMAF** form (440 nm) gave the maximum fluorescence intensity at pH 3.00. Excitation at the maximum absorption wavelengths of the neutral, monoanionic, and dianionic forms (495, 503, and 510 nm, respectively) led to the highest fluorescence intensity at both pH 3.97 and 4.97 (Figure 4).

The quantum yield at pH 4.55, where the neutral form of **BBDMAF** is predominant, was 0.78, which is slightly lower than that for the neutral form of **BDMAF** (0.85) [9]. In contrast, the quantum yields of the cationic, monoanionic, and dianionic forms were 0.11, 0.013 and 0.00080, respectively (Table S1). We then compared the radiative and nonradiative decay rate constants of neutral and monoanionic **BBDMAF**. The radiative decay rate was 41-fold higher for the neutral form than for the monocationic form, but the nonradiative decay rate was 6.5-fold lower. This indicated that the dramatic increase in the quantum yield of the neutral form of **BBDMAF** results from the enhancement of radiative decay and suppression of nonradiative decay (Figure S3).

Similar to **BDMAF**, the analysis of the pH-dependent emission behavior of **BBDMAF** revealed a significant reduction in the emission intensities at pH > 6 due to PET caused by the lone pair of electrons on the amino groups [9,18]. Instead, between pH 3 and 6, where protonation of the amino groups of **BBDMAF** occurs to some extent, PET was suppressed and very strong fluorescence was observed. The fluorescence of **BBDMAF**, which was expected to increase at pH < 3 due to further suppression of PET, decreased due to the formation of the ammonium xanthene form, which shows very low absorption. In addition, the absorbance of **BBDMAF** increased in the pH region where the cationic xanthene form (dicationic form) prevailed because its absorption is known to be greater than that of the ammonium xanthene form [9,19,20]. Accordingly, we measured the absorption and emission spectra of **BDMAF** and **BBDMAF** at pH −0.30, −0.56, and −0.94 (Figure S4). As expected, the absorbance increased upon decreasing the pH. However, the fluorescence intensities of the dicationic forms were quenched for **BDMAF** or decreased for **BBDMAF** when the pH was lowered, suggesting that the ammonium xanthene and cationic xanthene forms of **BBDMAF**, present at pH < 3, displayed very low emission. Notably, this pH-dependent fluorescence behavior of **BBDMAF** under illumination with a transilluminator is readily observable with the naked eye, as shown in Figure 4b.

### 2.4. Suzuki and Sonogashira Reactions of **BBDMAF**

**Scheme 3:**
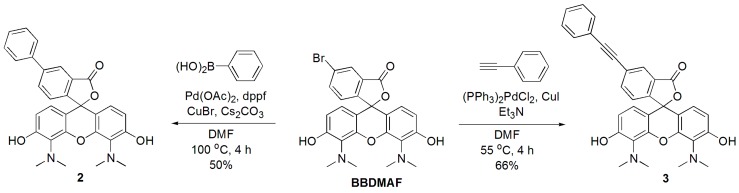
Suzuki and Sonogashira reactions of **BBDMAF**. DMF: *N,N*-dimethylformamide.

The suitability of **BBDMAF** as a starting material for cross-coupling reactions was evaluated against the Suzuki and Sonogashira couplings, and the desired products **2** and **3** were obtained in 50% and 66% yield, respectively (Scheme 3) [21,22]. Therefore, it is assumed that **BBDMAF** can be employed in various cross-coupling reactions if the appropriate reaction conditions are used.

**Figure 5:**
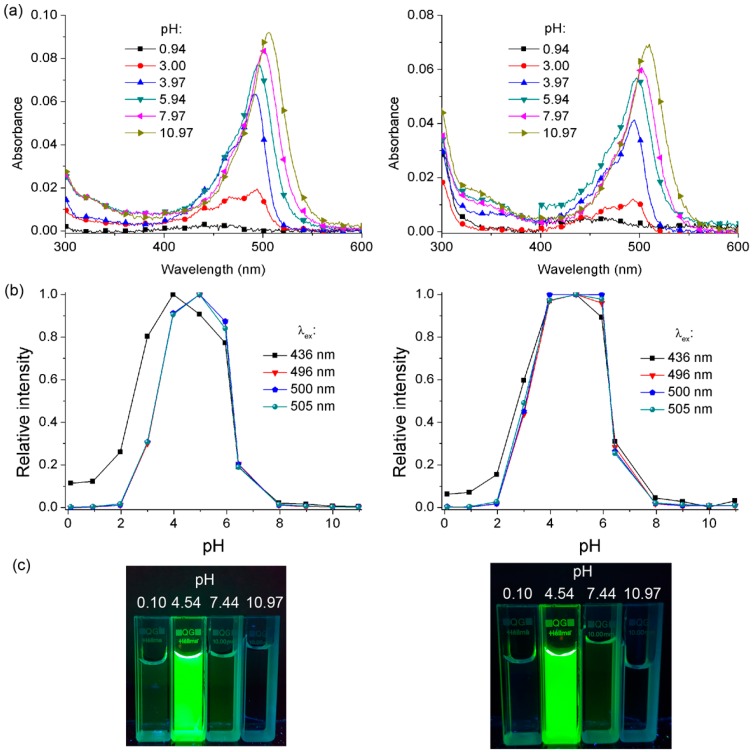
(**a**) Selected absorption spectra of **2** (2.5 μM, left) and **3** (2.5 μM, right) in 10 mM phosphate buffer at several pH levels at 25 °C. The spectra at pH 0.94 were obtained in HCl solution. All samples contain 0.05% DMSO to ensure solubility. (**b**) Relative intensity of maximum emission of **2** (512 nm, left) and **3** (514 nm, right), plotted with respect to pH; each protolytic species was excited at its maximum absorption wavelength. (**c**) Photographs of **2** (left) and **3** (right) solutions (concentration of each: 2.5 μM) illuminated under a UV transilluminator (312 + 365 nm).

In order to assess whether the photophysical properties of **BBDMAF** were maintained in compounds **2** and **3**, their pH-dependent absorbance and protolytic equilibria were investigated (Figure 5a, Figures S5 and S6). As shown in Table 1, the absorption behavior and p*K*_a_ values of compounds **2** and **3** resemble those of **BBDMAF** (Table 1). In addition, compounds **2** and **3** exhibited maximum emissions at 512 and 514 nm, respectively, regardless of the excitation wavelength (Figures S7 and S8), and exhibited strong fluorescence only at pH 3–6, as observed for **BDMAF** and **BBDMAF** (Figure 5b,c). These results indicate that the products of the cross-coupling reaction of **BBDMAF** retain the characteristic pH-dependent photophysical properties of **BDMAF** and **BBDMAF**.

## 3. Materials and Methods 

### 3.1. General

Commercially available reagents were used without further purification. Regioisomerically pure 5-bromofluorescein diacetate **1** was synthesized using a previously reported procedure [13]. Infrared (IR) spectra were recorded using a JASCO FT/IR-4100 spectrometer (Jasco Corporation, Tokyo, Japan). ^1^H and ^13^C NMR spectra were recorded using an AVANCE III 500 MHz NMR spectrometer (Bruker Biospin, Karlsruhe, Baden-Württemberg, Germany). High-resolution electron ionization (EI) and fast atom bombardment (FAB) mass spectra were recorded using a JEOL JMS-700 mass spectrometer (JEOL Ltd., Tokyo, Japan) at the Daegu Center of KBSI, Korea (Figures S9–S18).

### 3.2. Synthetic Procedures

#### 3.2.1. 5-Bromo-4′,5′-dinitrofluorescein (**BDNF**)

Sulfuric acid (32 mL) was added to 5-bromofluorescein diacetate **1** (1.0 g, 2.0 mmol), and the resulting mixture was stirred at −5 °C until **1** dissolved completely. After addition of nitric acid (2.3 mL), the mixture was stirred for a further 20 min at −5 °C. The mixture was then poured into cold water and extracted with EtOAc. The extracts were combined and dried over Na_2_SO_4_. After evaporation of the solvent under vacuum, the residue was purified by column chromatography on silica gel (CH_2_Cl_2_/MeOH = 4:1) to afford **BDNF** as an orange solid, which was recrystallized from EtOAc/hexane (1:2) (0.81 g, 80%); M.p. > 158 °C dec.; IR (film): *ν* 3422, 1767, 1619, 1533, 1451, 1370, 1284, 1246, 1223, 1087, 1000, 950, 885, 820 cm^−1^; ^1^H NMR (500 MHz, DMSO-*d*_6_): *δ* 11.95 (s, 2H; OH), 8.25 (d, *J* =1.37 Hz, 1H; H-4), 8.03 (dd, *J* = 8.24, 1.83 Hz, 1H; H-6), 7.55 (d, *J* = 7.6, 0.8 Hz, 1H; H-7), 6.98 (d, *J* = 9.0 Hz, 2H; H-1′, H-8′), 6.88 (d, *J* = 9.0 Hz, 2H; H-2′, H-7′); ^13^C NMR (125 MHz, DMSO-*d*_6_): *δ* 167.1, 152.2, 150.5, 142.5, 139.3, 131.4, 129.0, 128.7, 128.1, 127.1, 124.4, 114.7, 110.0, 80.9; HRMS–EI (*m*/*z*): [M]^+^ calcd for C_20_H_9_BrN_2_O_9_, 499.9491; found, 499.9495. 

#### 3.2.2. 5-Bromo-4′,5′-diaminofluorescein (**BDAF**)

Tin chloride (4.5 g, 20 mmol) was added to a solution of **BDNF** (1.0 g, 2.0 mmol) in ethanol (70 mL), and the mixture was then refluxed for 6 h. After evaporation of the solvent under vacuum, 1 M NaOH (20 mL) was added, and the mixture was filtered to remove tin chloride. The filtrate was washed with water. The solution was acidified with 1 M HCl to pH 4–5 and extracted with EtOAc. The combined extracts were dried (Na_2_SO_4_), and the solvent was removed under vacuum to afford **BDAF** as a purple solid, which was recrystallized from hot acetone (0.62 g, 70%); M.p. > 400 °C dec.; IR (film): *ν* 3459, 3344, 1754, 1628, 1564, 1511, 1463, 1410, 1347, 1284, 1252, 1156, 1011, 950, 858, 768, 702 cm^−1^; ^1^H NMR (500 MHz, DMSO-*d*_6_): *δ* 9.58 (s, 2H; OH), 8.12 (d, *J* = 1.53 Hz, 1H; H-4), 7.94 (dd, *J* = 8.24, 1.83 Hz, 1H; H-6), 7.20 (d, *J* = 8.09 Hz, 1H; H-7), 6.49 (d, *J* = 8.39 Hz, 2H; H-1′, H-8′), 5.78 (d, *J* = 8.55 Hz, 2H; H-2′, H-7′), 5.10 (s, 4H; NH); ^13^C NMR (125 MHz, DMSO-*d*_6_): *δ* 167.8, 152.0, 145.2, 139.6, 138.4, 129.2, 127.5, 126.8, 125.0, 123.2, 113.8, 111.1, 110.4, 85.6; HRMS–EI (*m*/*z*): [M]^+^ calcd for C_20_H_13_BrN_2_O_5_, 440.0008; found, 440.0011.

#### 3.2.3. 5-Bromo-4′,5′-bis(dimethylamino)fluorescein (**BBDMAF**)

A formaldehyde solution (37 wt % in H_2_O, 1.76 mL, 24 mmol) was added to a solution of **BDAF** (1.0 g, 2.2 mmol) in methanol (50 mL), and the resulting mixture was stirred at room temperature for 15 min. After the addition of NaBH_3_CN (1.7 g, 27 mmol), the mixture was stirred for 14 h at room temperature. The mixture was then concentrated under vacuum, and water (100 mL) was added. The solution was acidified with 1 M HCl to pH 4–5 and extracted with CH_2_Cl_2_. The extracts were combined and dried over Na_2_SO_4_. The solvent was removed under vacuum, and the residue was purified by column chromatography on silica gel (CH_2_Cl_2_/MeOH = 10:1) to afford **BBDMAF** as an orange solid, which was recrystallized from CH_2_Cl_2_/hexane (1:2) (0.57 g, 50%); M.p. > 241 °C dec.; IR (film): *ν* 2912, 2850, 1759, 1591, 1490, 1425, 1302, 1259, 1203, 1139, 1129, 1099, 1045, 936, 857, 783, 808, 715 cm^−1^; ^1^H NMR (500 MHz, DMSO-*d*_6_): *δ* 9.44 (s, 2H; OH), 8.16 (d, *J* = 1.37 Hz, 1H; H-4), 7.96 (dd, *J* = 8.09, 1.83 Hz, 1H; H-6), 7.34 (d, *J* = 8.24 Hz, 1H; H-7), 6.60 (d, *J* = 8.85 Hz, 2H; H-1′, H-8′), 6.40 (d, *J* = 8.70 Hz, 2H; H-2′, H-7′), 2.91 (s, 12H; CH_3_); ^13^C NMR (125 MHz, DMSO-*d*_6_): *δ* 167.5, 157.1, 151.3, 150.3, 138.6, 129.3, 127.7, 127.1, 126.9, 125.0, 123.6, 112.3, 110.2, 85.1, 43.7; HRMS–EI (*m*/*z*): [M]^+^ calcd for C_24_H_21_BrN_2_O_5_, 496.0634; found, 496.0631.

#### 3.2.4. 5-Phenyl-4′,5′-bis(dimethylamino)fluorescein (**2**)

A solution of **BBDMAF** (200 mg, 0.39 mmol), phenylboronic acid (74 mg, 0.60 mmol), Pd(OAc)_2_ (9.0 mg, 0.039 mmol), CuBr (58 mg, 0.39 mmol), Cs_2_CO_3_ (52 mg, 1.6 mmol), and dppf (45 mg, 0.080 mmol) in *N*,*N*-dimethylformamide (DMF) (6.0 mL) was degassed by bubbling argon through the solution. The mixture was then heated at 100 °C for 4 h. After evaporation of the solvent under vacuum, water (50 mL) was added. The solution was acidified with 1 M HCl to pH 4–5 and extracted with EtOAc. The combined extracts were dried (Na_2_SO_4_). The solvent was evaporated under vacuum, and the residue was purified by column chromatography (CH_2_Cl_2_/MeOH = 10:1) (99 mg, 50%); M.p. > 110 °C dec.; IR (film): *ν* 3264, 2922, 2852, 1760, 1594, 1491, 1464, 1424, 1307, 1201, 1138, 1098, 1067, 1053, 1023, 875, 812, 785, 760 cm^−1^; ^1^H NMR (500 MHz, DMSO-*d*6): *δ* 9.45 (s, 2H; OH), 8.18 (d, *J* =1.1 Hz, 1H; H-4), 8.09 (dd, *J* = 8.1, 1.7 Hz, 1H; H-6), 7.83 (dd, *J* = 8.4, 1.2 Hz, 2H; PhH), 7.54 (t, *J* = 7.3 Hz, 2H; PhH), 7.48‒7.43 (m, 2H; H-7, PhH), 6.61 (d, *J* = 8.7 Hz, 2H; H-1′, H-8′), 6.42 (d, *J* = 8.7 Hz, 2H; H-2′, H-7′); HRMS–FAB (*m*/*z*): [M + H]^+^ calcd for C_30_H_27_N_2_O_5_, 495.1920; found, 495.1923.

#### 3.2.5. 5-Phenylethynyl-4′,5′-bis(dimethylamino)fluorescein (**3**)

**BBDMAF** (200 mg, 0.40 mmol), phenylacetylene (132 µL, 1.2 mmol), Pd(PPh_3_)_2_Cl_2_ (28 mg, 0.040 mmol), and CuI (8 mg, 0.040 mmol) were dissolved in DMF (2.1 mL) and triethylamine (0.7 mL). After bubbling argon through the solution for 2 min, the mixture was subjected to 10 pump/purge cycles and then stirred at 55 °C for 4 h. The solvent was evaporated under vacuum, and the residue was purified by column chromatography (CH_2_Cl_2_/MeOH = 40:1) (460 mg, 66%); M.p. > 120 °C dec.; IR (film): *ν* 2953, 2922, 2853, 1768, 1739, 1622, 1453, 2395, 1377, 1320, 1242, 1216, 1122, 1060, 980, 894, 828, 804 cm^−1^; ^1^H NMR (500 MHz, CD_3_OD): *δ* 8.06 (s, 1H; H-4), 7.38 (dd, *J* = 7.8, 1.1 Hz, 1H; H-6), 7.48 (dd, *J* = 6.0, 2.3 Hz, 2H; phH), 7.34–7.31 (m, 3H; phH), 7.24 (d, *J* = 7.9 Hz, 1H; H-7), 6.87‒6.80 (m, 4H; H-1′, H-2′, H-7′, H-8′), 3.46 (s, 12H; CH_3_).; HRMS–FAB (*m*/*z*) : [M + H]^+^ calcd for C_32_H_27_N_2_O_5_, 519.1920; found 519.1918.

### 3.3. Buffer Solutions

The pH range investigated was between 0.00 and 10.97. A 35% HCl solution was used to prepare solutions with pH values below 1.45. The Henderson–Hasselbach equation was used to calculate the amount of acid and base needed to prepare the different phosphate buffer solutions. These solutions were prepared by mixing phosphoric acid and sodium phosphate monobasic (pH 1.97–4.55), and sodium phosphate monobasic and sodium phosphate dibasic (pH 4.97–10.97). The pH was adjusted by adding 1 M NaOH or 1 M HCl, and measured using a WTW inoLab pH 720 pH meter (WTW GmbH, Weilheim, Germany). The ionic strength was controlled using a 0.16 M NaCl solution. All samples were prepared from a stock solution in DMSO to ensure solubility; hence, all samples contained 0.05% DMSO.

### 3.4. UV–Vis and Fluorescence Spectroscopy

UV–Vis spectra were recorded at 25 °C in a 10-mm path quartz cell using a Cary 100 UV–Vis spectrophotometer (Agilent, Santa Clara, CA, USA) with pure solvent as a reference. Fluorescence spectra were recorded at 25 °C using a Cary Eclipse fluorescence spectrophotometer (Agilent, Santa Clara, CA, USA) (cell path length: 1 cm). The fluorescence quantum yields (*Φ*_F_) were determined using a 0.1-M aqueous NaOH solution of fluorescein as a standard [23]. The p*K*_a_ values were determined by the DATAN program, using the physical constraints approach [15,16,17].

### 3.5. Fluorescence Lifetime Measurement

A time-correlated single photon counting (TCSPC) system for fluorescence lifetime (*τ*_F_) measurement has been described elsewhere [9]. In brief, the apparatus consisted of a picosecond 445 nm diode laser (Edinburgh Instrument, , Livingston, West Lothian, UK), a TCSPC PCI board (SP130, Becker & Hickl GmbH, Berlin, Germany), and an avalanche photodiode (ID Quantique, Geneva, Switzerland). On the basis of the fluorescence lifetime and quantum yield, the radiative (*k*_r_) and nonradiative rate constants (*k*_nr_) were calculated according to the following equations: *k*_r_ = *Φ*_F_/*τ*_F_ and *k*_nr_ = (1 − *Φ*_F_)/*τ*_F_.

## 4. Conclusions

In conclusion, three new fluorescein derivatives—**BDNF**, **BDAF** and **BBDMAF**—were synthesized from 5-bromofluorescein diacetate through a series of standard reactions. Their pH-dependent protolytic equilibria and p*K*_a_ values were determined using UV‒Vis spectra. Among these derivatives, only **BBDMAF** showed strong emission in a specific pH range (pH 3–6). Moreover, **BBDMAF** could be functionalized via the Suzuki and Sonogashira cross-coupling reactions. This unique fluorescence property of **BBDMAF** is thought to be useful for preparing a wide range of derivatives via the cross-coupling reaction that can be used as sensors and biomarkers.

## Figures and Tables

**Table 1:** Acidity constants of fluorescein derivatives ^1^.

Compound	p*K*_a1_	p*K*_a2_	p*K*_a3_
**DNF** ^2^	5.36	5.00	n.d. ^3^
**BDNF**	3.19	3.22	n.d. ^3^
**DAF** ^2^	2.68	4.27	6.40
**BDAF**	2.37	3.99	6.51
**BDMAF** ^2^	3.37	5.83	8.68
**BBDMAF**	2.96	5.77	9.50
**2**	3.42	5.87	9.02
**3**	3.52	5.53	8.84

^1^ The p*K*_a_ values were calculated using the DATAN program. ^2^ Reported p*K*_a_ values [3,6]. ^3^ Not detectable.

## Supplementary Materials

The following are available online. Figure S1: absorption spectra, Table S1: photophysical properties, Figure S2: emission spectra, Figure S3: fluorescence decay profiles, Figure S4: absorption and emission spectra in HCl solution, Figures S5–S8: spectra of **2** and **3**, Figures S9–S18: ^1^H NMR and HRMS spectra.
