# Supplementary material for: 5-Bromo-4′,5′-bis(dimethylamino)fluorescein: Synthesis and Photophysical Studies
Source: Molecules. 2018 Jan 20;23(1):219. doi: 10.3390/molecules23010219 (PMC6017165; doi:10.3390/molecules23010219)
Supplement: Supplementary file 1 [file molecules-23-00219-s001.docx]

Supplementary Materials for

**5-Bromo-4',5'-bis(dimethylamino)fluorescein: Synthesis and Photophysical Studies**

Jun Yeon Hwang ^1^, Jung-Yean Lee ^1^, Chang-Woo Cho ^1^, Wonjun Choi ^2^, Yejin Lee ^2^, Sangdeok Shim ^2,^* and Gil Tae Hwang ^1,^*

^1^ Department of Chemistry and Green-Nano Materials Research Center, Kyungpook National University, Daegu 41566, Republic of Korea; E-mail: giltae@knu.ac.kr;

^2^ Department of Chemistry, Sunchon National University, 255 Jungang-ro, Sunchon, Jeonnam 57922, Republic of Korea; san90@scnu.ac.kr

| **Figure S1**. Absorption spectra of **BDNF**, **BDAF**, and **BBDMAF** | S2 |
| --- | --- |
| **Table S1.** Photophysical properties of each protolytic species of **BDNF**, **BDAF**, and **BBDMAF** | S3 |
| **Figure S2.** Emission spectra of **BBDMAF** | S4 |
| **Figure S3**. Fluorescence decay profiles, fluorescence lifetime (*τ*_F_), the radiative (*k*_r_), and nonradiative rate constant of **BBDMAF** | S5 |
| **Figure S4.** Absorption and emission spectra of **BDMAF** and **BBDMAF** in HCl solution at pH ‒0.30, ‒0.56, and ‒0.94 at 25 °C | S6 |
| **Figure S5.** (a) Absorption spectra of **2**. (b) Proposed spectra for the protolytic forms of **2**. (c) Relative concentration curves for each protolytic species of **2** versus pH | S7 |
| **Figure S6.** (a) Absorption spectra of **3**. (b) Proposed spectra for the protolytic forms of **3**. (c) Relative concentration curves for each protolytic species of **3** versus pH | S8 |
| **Figure S7.** Emission spectra of **2** in 10 mM phosphate buffer at several pH values at 25 °C | S9 |
| **Figure S8.** Emission spectra of **3** in 10 mM phosphate buffer at several pH values at 25 °C | S10 |
| **Figure S9.** ^1^H NMR spectrum of **BDNF** in DMSO-*d*_6_ | S11 |
| **Figure S10.** HRMS-EI spectrum of **BDNF**. | S12 |
| **Figure S11.** ^1^H NMR spectrum of **BDAF** in DMSO-*d*_6_ | S13 |
| **Figure S12.** HRMS-EI spectrum of **BDAF**. | S14 |
| **Figure S13.** ^1^H NMR spectrum of **BBDMAF** in DMSO-*d*_6_ | S15 |
| **Figure S14.** HRMS-EI spectrum of **BBDMAF**. | S16 |
| **Figure S15.** ^1^H NMR spectrum of **2** in DMSO-*d*_6_ | S17 |
| **Figure S16.** HRMS-FAB spectrum of **2**. | S18 |
| **Figure S17.** ^1^H NMR spectrum of **3** in CD_3_OD | S19 |
| **Figure S18.** HRMS-FAB spectrum of **3**. | S20 |


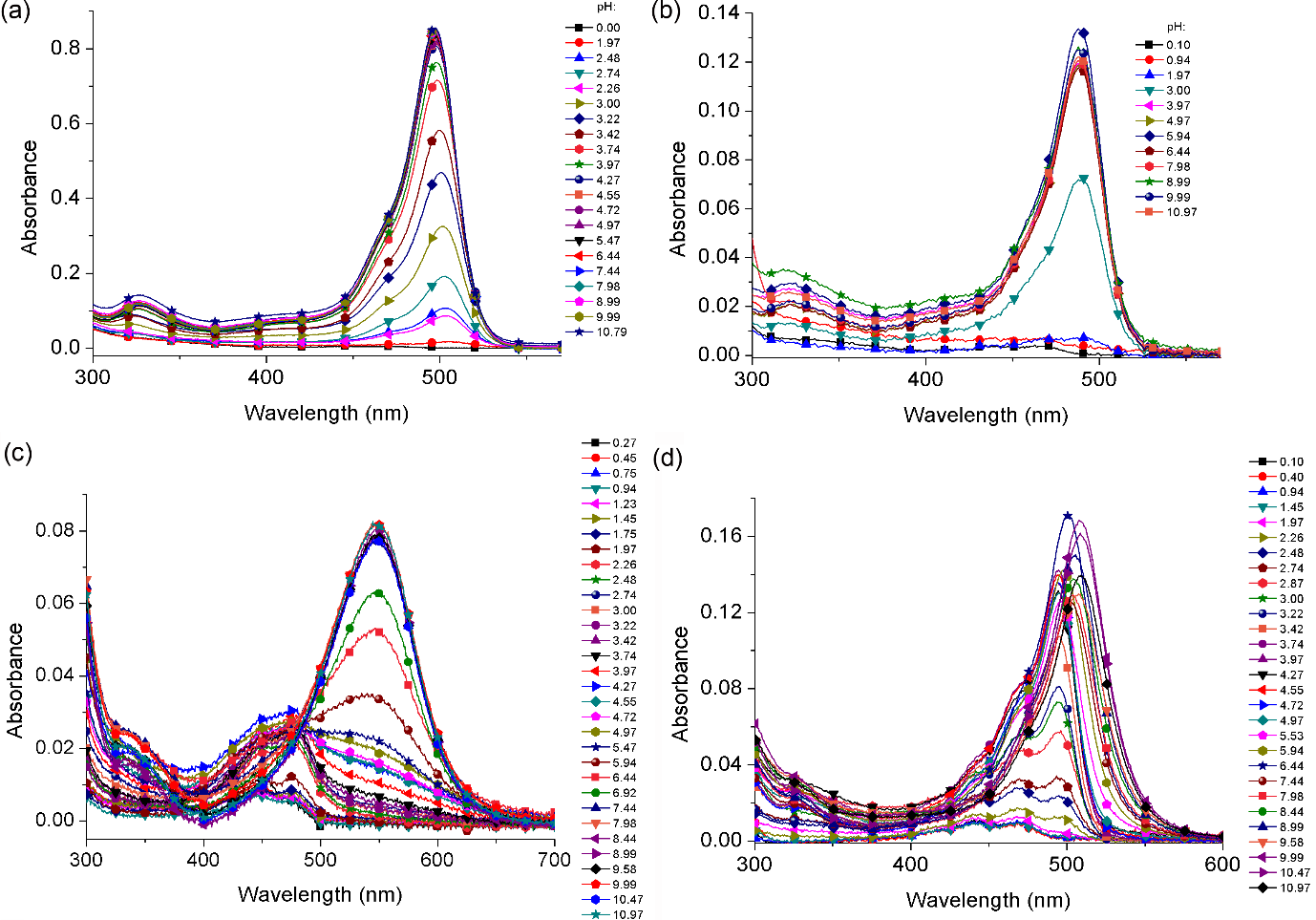


**Figure S1.** Absorption spectra of (a) **BDNF** (12.5 μM) in EtOH:10 mM phosphate buffer (1:1, v/v), (b) **BDNF** (2.5 μM), (c) **BDAF** (2.5 μM), and (d) **BBDMAF** (2.5 μM) in 10 mM phosphate buffer at several pH values at 25 °C. The spectra at pH 0.15‒1.45 were obtained in HCl solution. All samples contain 0.05% DMSO to ensure solubility.

**Table S1.** Photophysical properties of each protolytic species of **BDNF**, **BDAF**, and **BBDMAF**

| Dyes | Protolytic form ^1^ | *λ*_max_ (nm) ^2^ | *ε* (M^-1^cm^-1^) ^3^ | *λ*_em_ (nm) ^4^ | *Ф*_F_ ^3,5^ |
| --- | --- | --- | --- | --- | --- |
| **BDNF** | Neutral (1.45) | 506 | 277 | nd ^6^ | nd ^6^ |
|  | Monianion (3.74) | 499 | 52,100 | nd ^6^ | nd ^6^ |
|  | Dianion (5.94) | 497 | 64,700 | nd ^6^ | nd ^6^ |
| **BDAF** | Cation (0.20) | 448 | 6,210 | nd ^6^ | nd ^6^ |
|  | Neutral (3.22) | 478 | 15,000 | nd ^6^ | nd ^6^ |
|  | Monianion (5.53) | 500 | 12,400 | nd ^6^ | nd ^6^ |
|  | Dianion (9.58) | 545 | 37,500 | nd ^6^ | nd ^6^ |
| **BBDMAF** | Cation (0.10) | 440 | 4,380 | 510 | 0.11 ^7^ |
|  | Neutral (4.55) | 495 | 66,700 | 513 | 0.78 ^8^ |
|  | Monianion (7.49) | 503 | 64,000 | 515 | 0.013 ^8^ |
|  | Dianion (10.97) | 510 | 68,100 | 515 | 0.00080 ^8^ |

^1^ pH values are listed in parentheses where the fraction of each protolytic species of **BDNF**, **BDAF**, and **BBDMAF** was maximized. ^2^ Wavelength of maximum absorbance. ^3^ All values of molar absorptivity and quantum yields are averages from at least three measurements. ^4^ Wavelength of emission maximum when excited at the absorption maximum. ^5^ Quantum efficiencies using fluorescein in 0.1 M NaOH as a standard. ^6^ Fluorescence was too low to be measured. ^7^ *λ*_ex_ = 436 nm. ^8^ *λ*_ex_ = 496 nm.


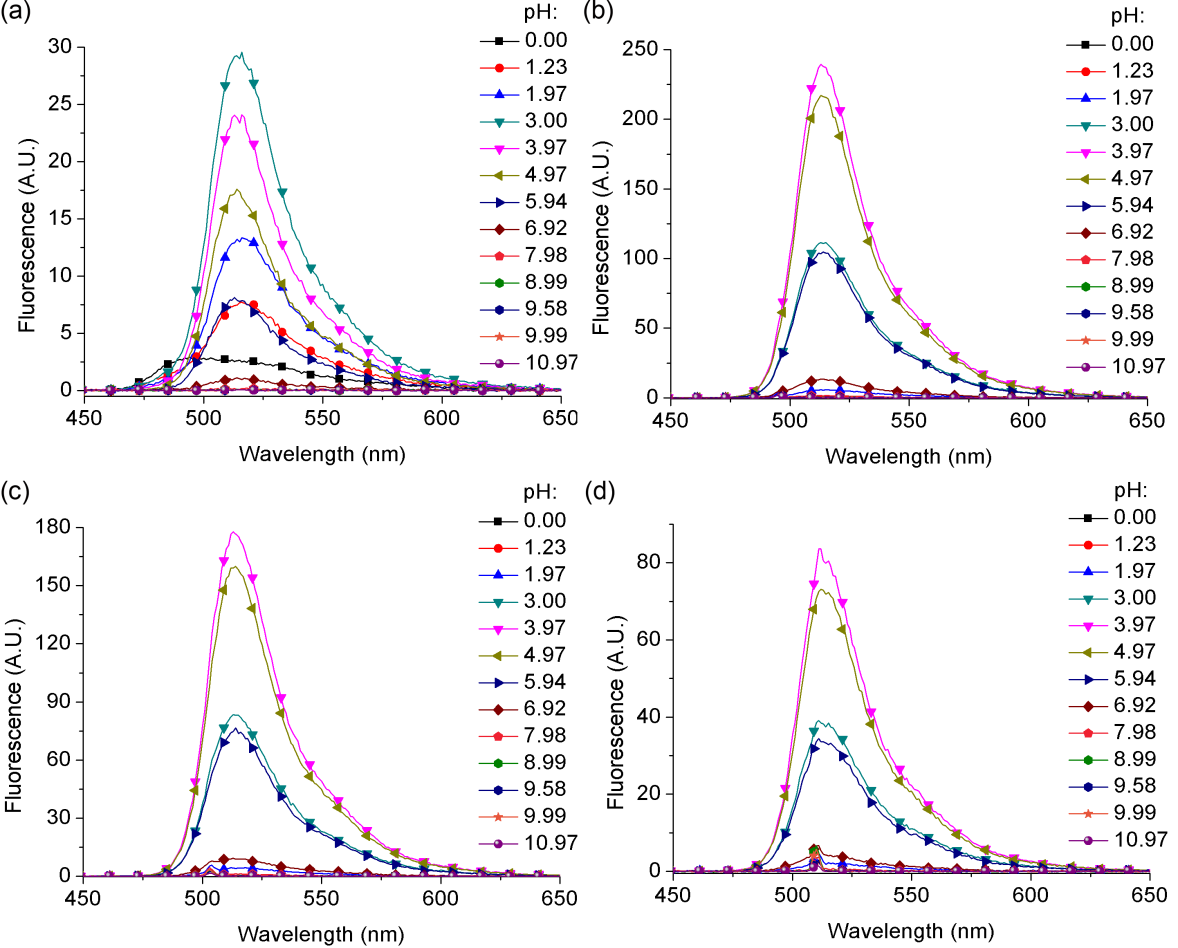


**Figure S2.** Emission spectra of **BBDMAF** (2.5 μm) in 10 mM phosphate buffer at several pH values at 25 °C. The spectra at pH 0.00 and 1.23 were obtained in HCl solution. All samples contain 0.05% DMSO to ensure solubility. Excited at (a) 440, (b) 495, (c) 503, and (d) 510 nm.


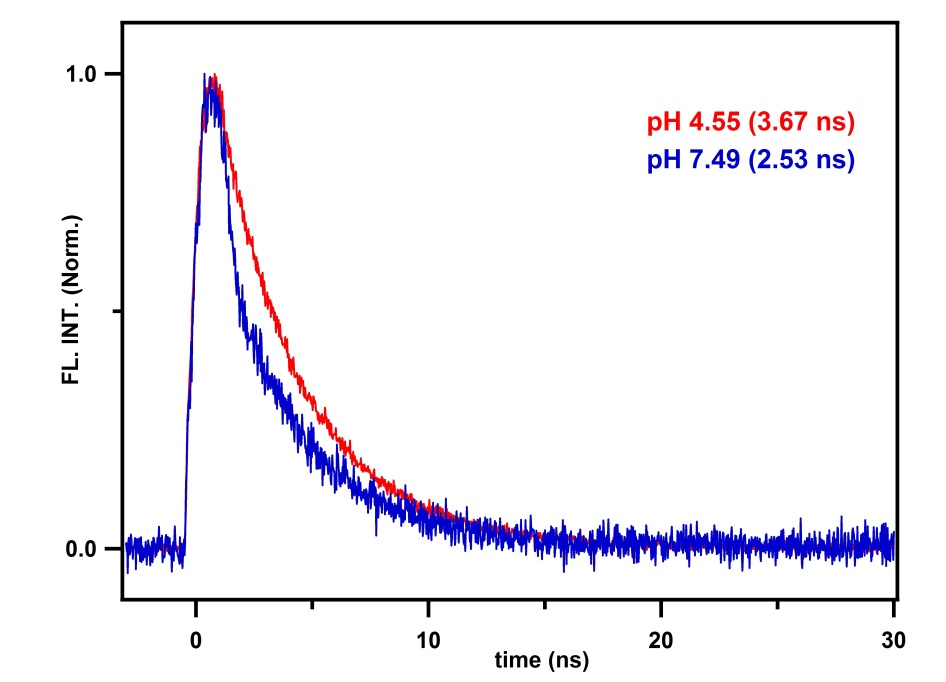


| Dye | Protolytic form ^1^ | *τ*_F_ (ns) | *k*_r_ (×10^7^ s^-1^) | *k*_nr_ (×10^7^ s^-1^) |
| --- | --- | --- | --- | --- |
| **BBDMAF** | Neutral (4.55) | 3.67 | 21.3 | 5.99 |
|  | Monianion (7.49) | 2.53 | 0.514 | 39.0 |

^1^ pH values are listed in parentheses where the fraction of each protolytic species of **BBDMAF** was maximized.

**Figure S3**. Fluorescence decay profiles, fluorescence lifetime (*τ*_F_), the radiative (*k*_r_), and nonradiative rate constant of **BBDMAF**.


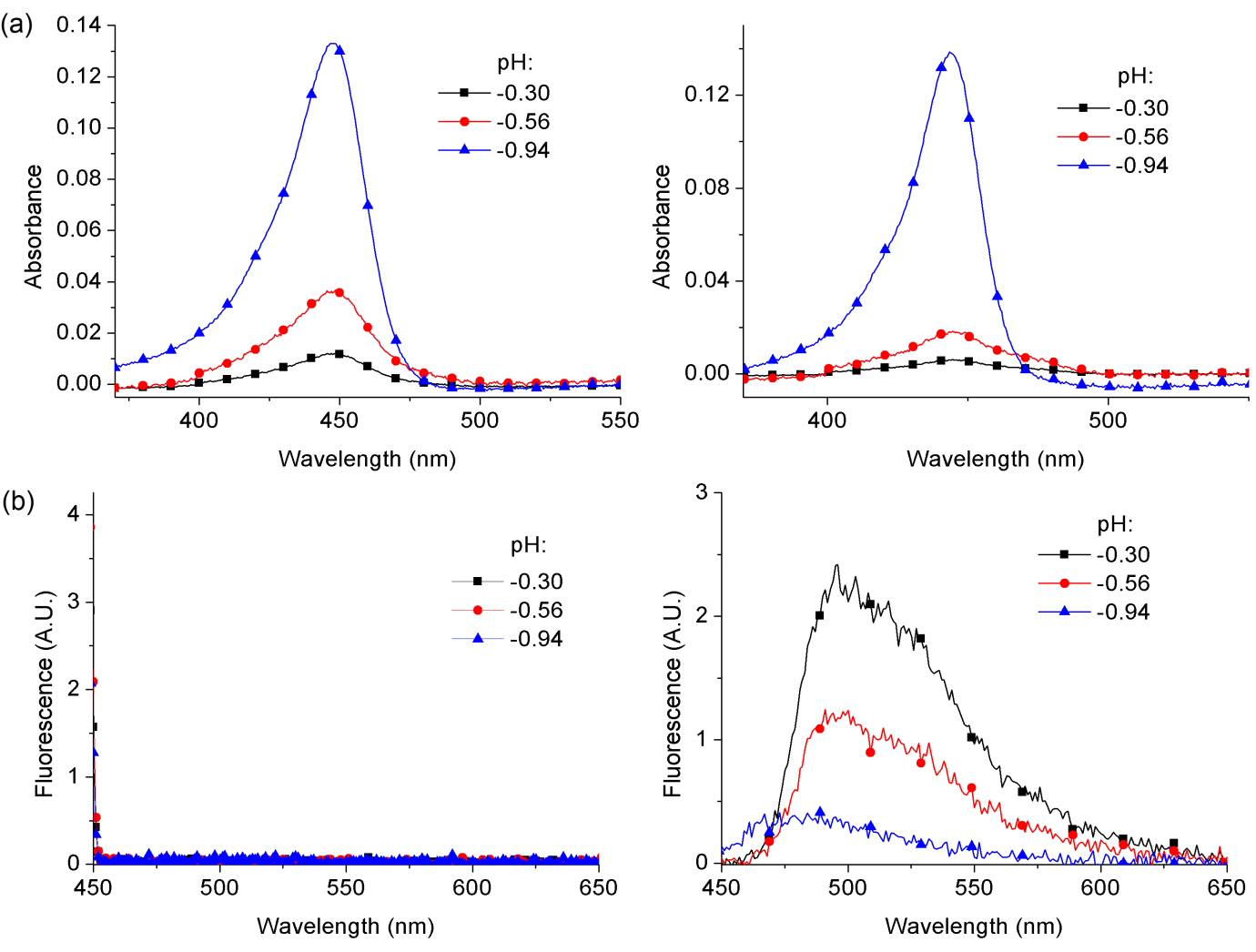


**Figure S4.** (a) Absorption and (b) emission spectra of **BDMAF** (2.5 μm, *λ*_ex_ = 448 nm, left) and **BBDMAF** (2.5 μm, *λ*_ex_ = 440 nm, right) in HCl solution at pH ‒0.30, ‒0.56, and ‒0.94 at 25 °C. All samples contained 0.05% DMSO to ensure solubility.


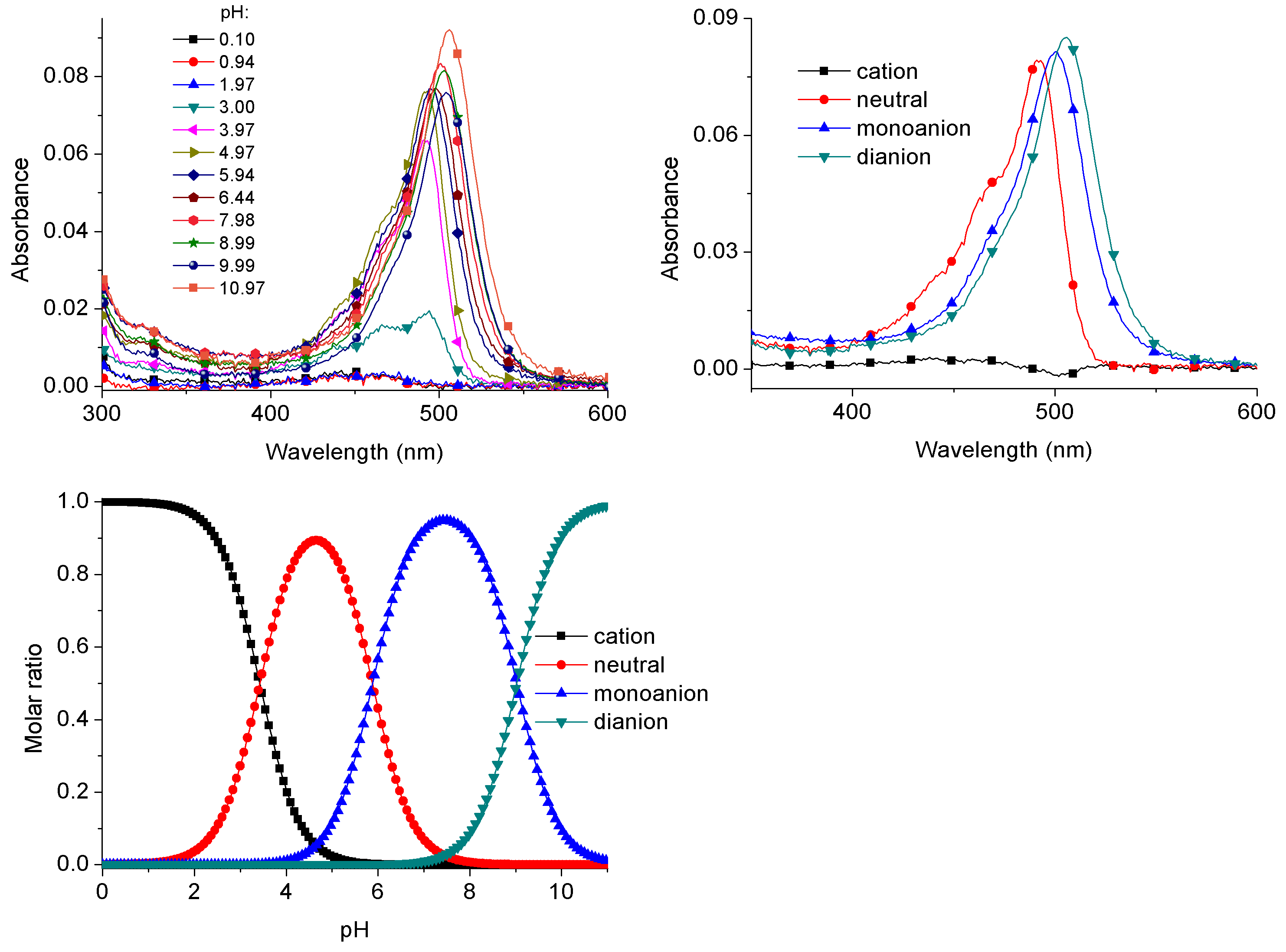


**Figure S5.** (a) Absorption spectra of **2** (2.5 μM) in 10 mM phosphate buffer at several pH values at 25 °C. The spectra at pH 0.10 and 0.94 were obtained in HCl solution. All samples contain 0.05% DMSO to ensure solubility. (b) Proposed spectra for the protolytic forms (cation, neutral, monoanion, and dianion) of **2** generated by the DATAN program. (c) Relative concentration curves for each protolytic species of **2** versus pH.


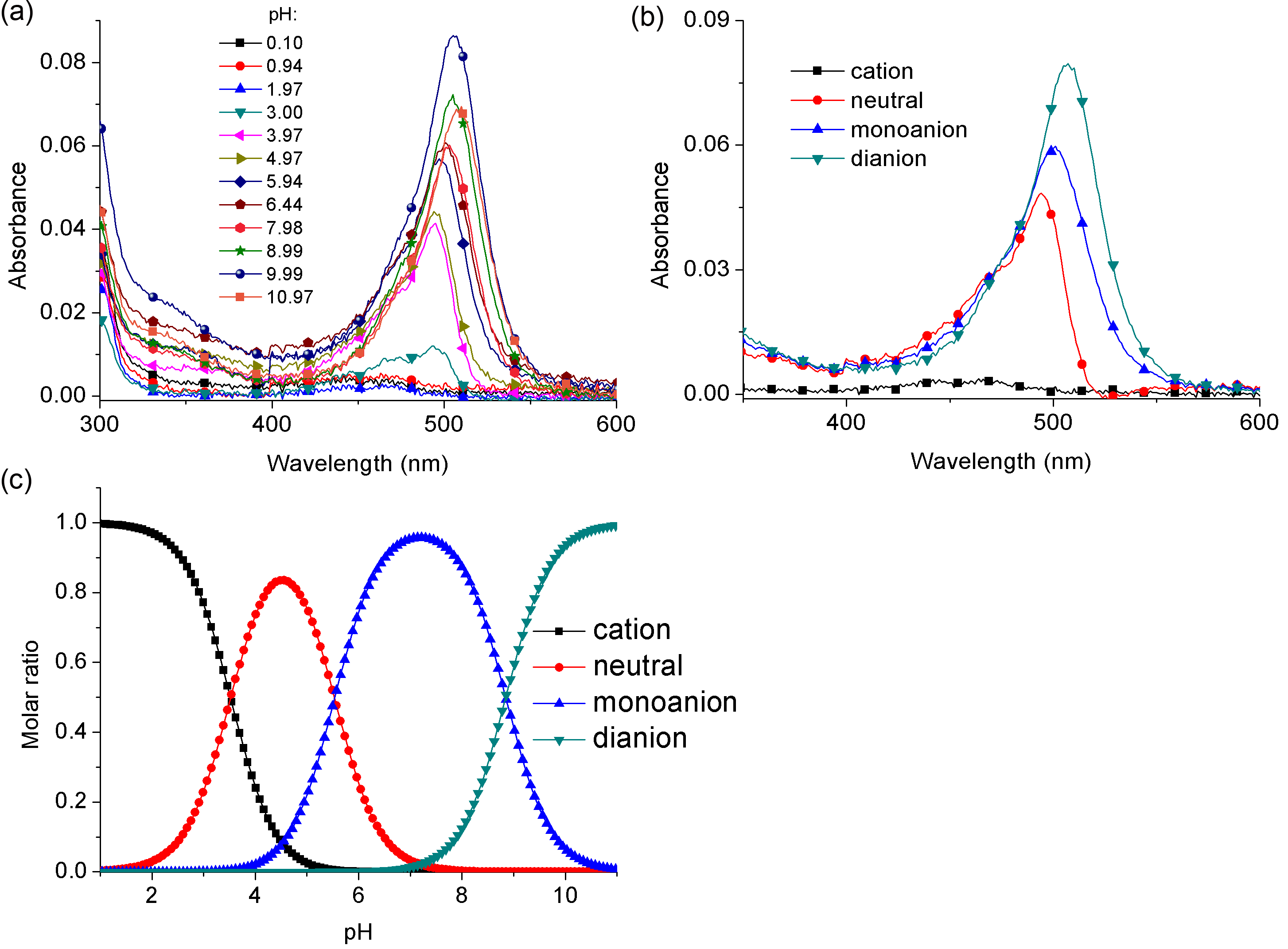


**Figure S6.** (a) Absorption spectra of **3** (2.5 μM) in 10 mM phosphate buffer at several pH values at 25 °C. The spectra at pH 0.10 and 0.94 were obtained in HCl solution. All samples contain 0.05% DMSO to ensure solubility. (b) Proposed spectra for the protolytic forms (cation, neutral, monoanion, and dianion) of **3** generated by the DATAN program. (c) Relative concentration curves for each protolytic species of **3** versus pH.


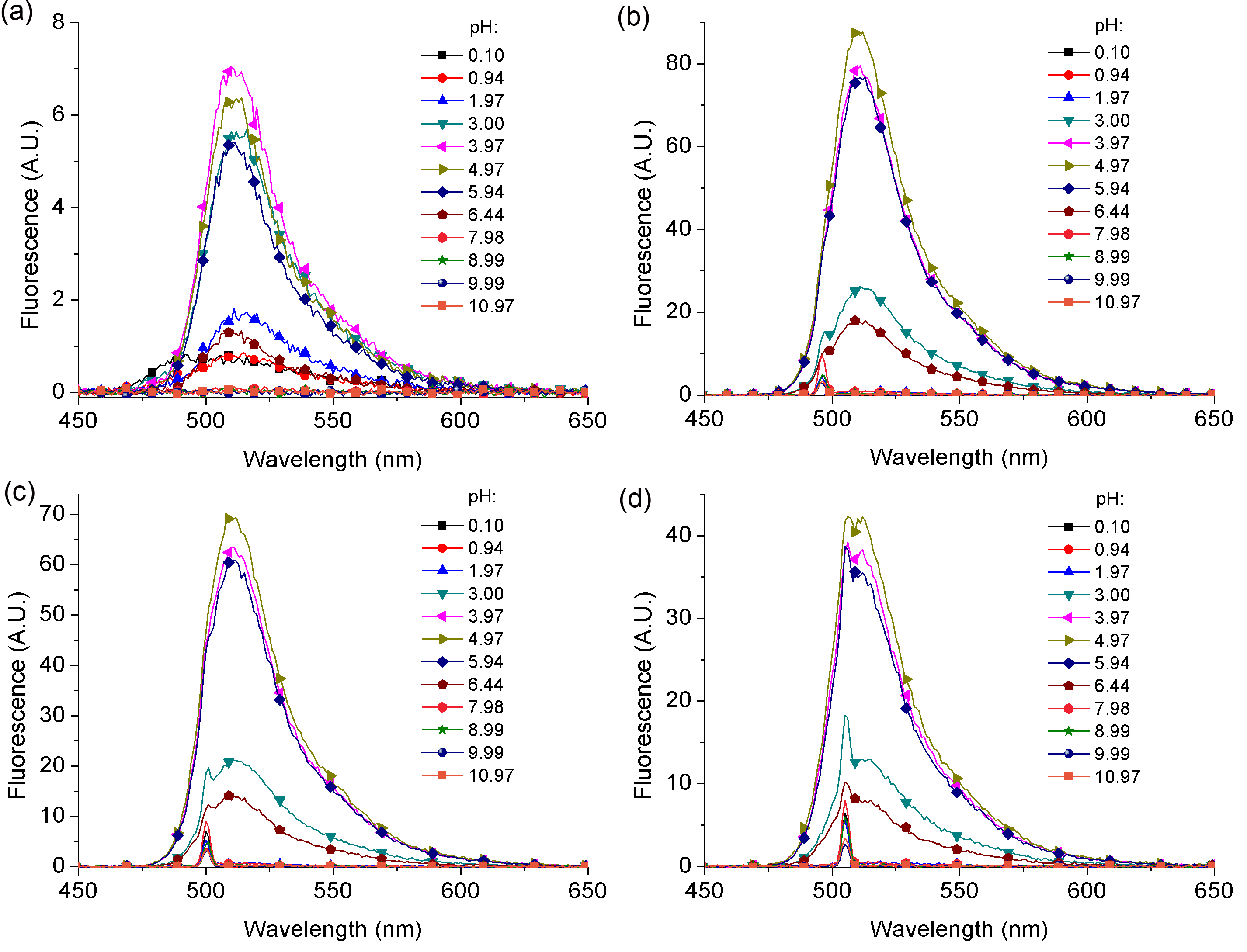


**Figure S7.** Emission spectra of **2** (2.5 μm) in 10 mM phosphate buffer at several pH values at 25 °C. The spectra at pH 0.10 and 0.94 were obtained in HCl solution. All samples contain 0.05% DMSO to ensure solubility. Excited at (a) 436, (b) 496, (c) 500, and (d) 505 nm.


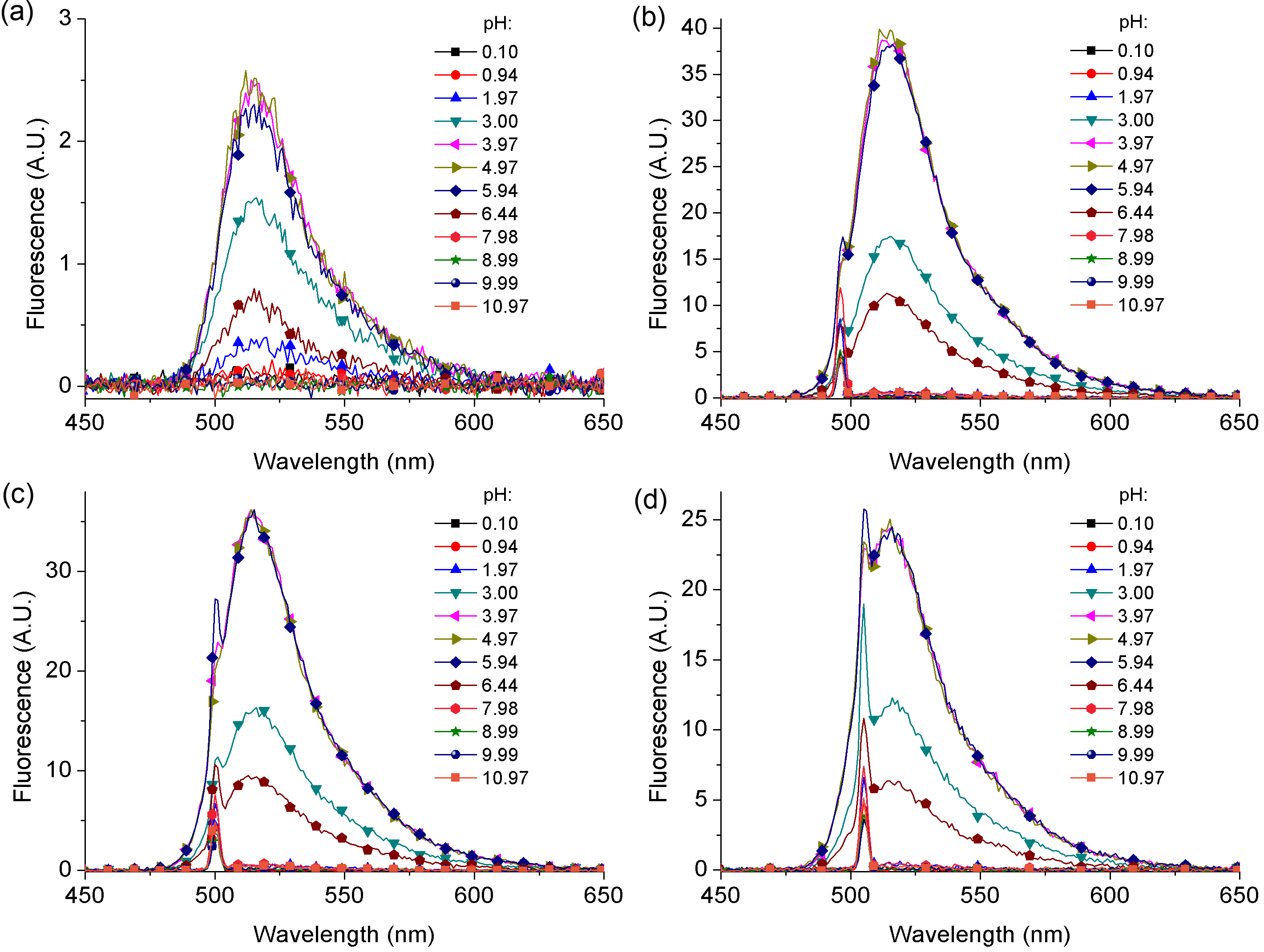


**Figure S8.** Emission spectra of **3** (2.5 μm) in 10 mM phosphate buffer at several pH values at 25 °C. The spectra at pH 0.10 and 0.94 were obtained in HCl solution. All samples contain 0.05% DMSO to ensure solubility. Excited at (a) 436, (b) 496, (c) 500, and (d) 505 nm.

**
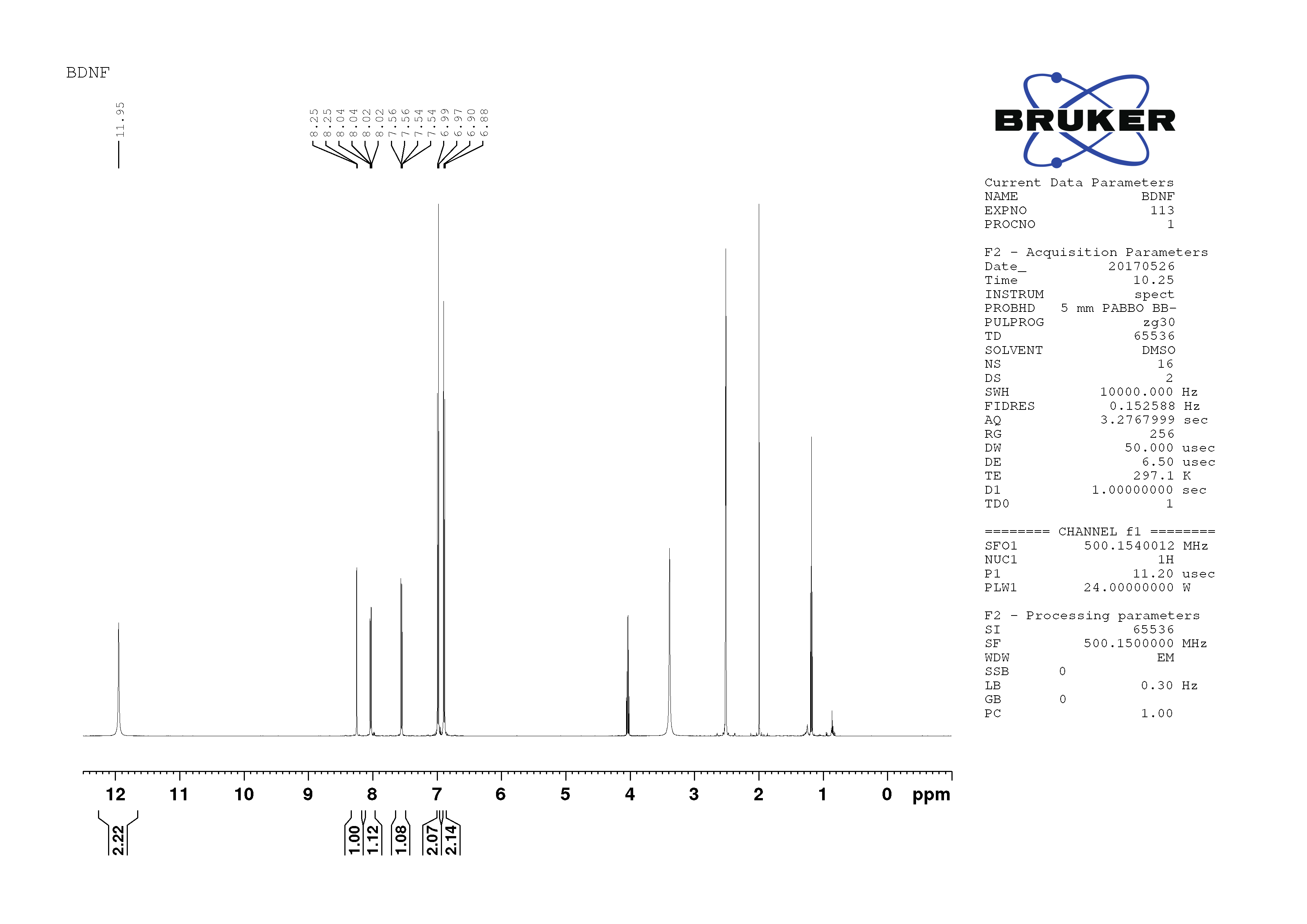
**

**Figure S9.** ^1^H NMR spectrum of **BDNF** in DMSO-*d*_6_.


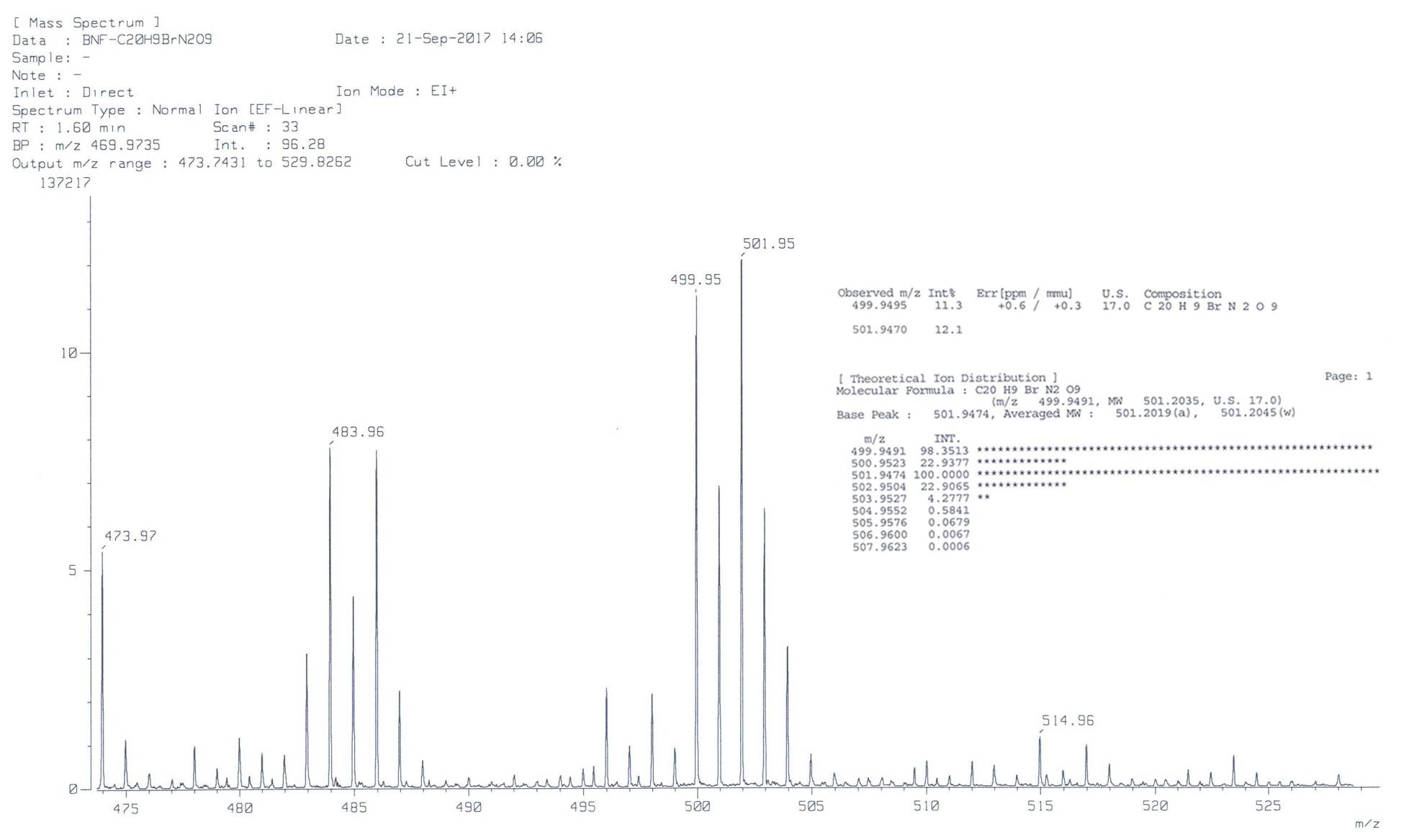


**Figure S10.** HRMS-EI spectrum of **BDNF**.


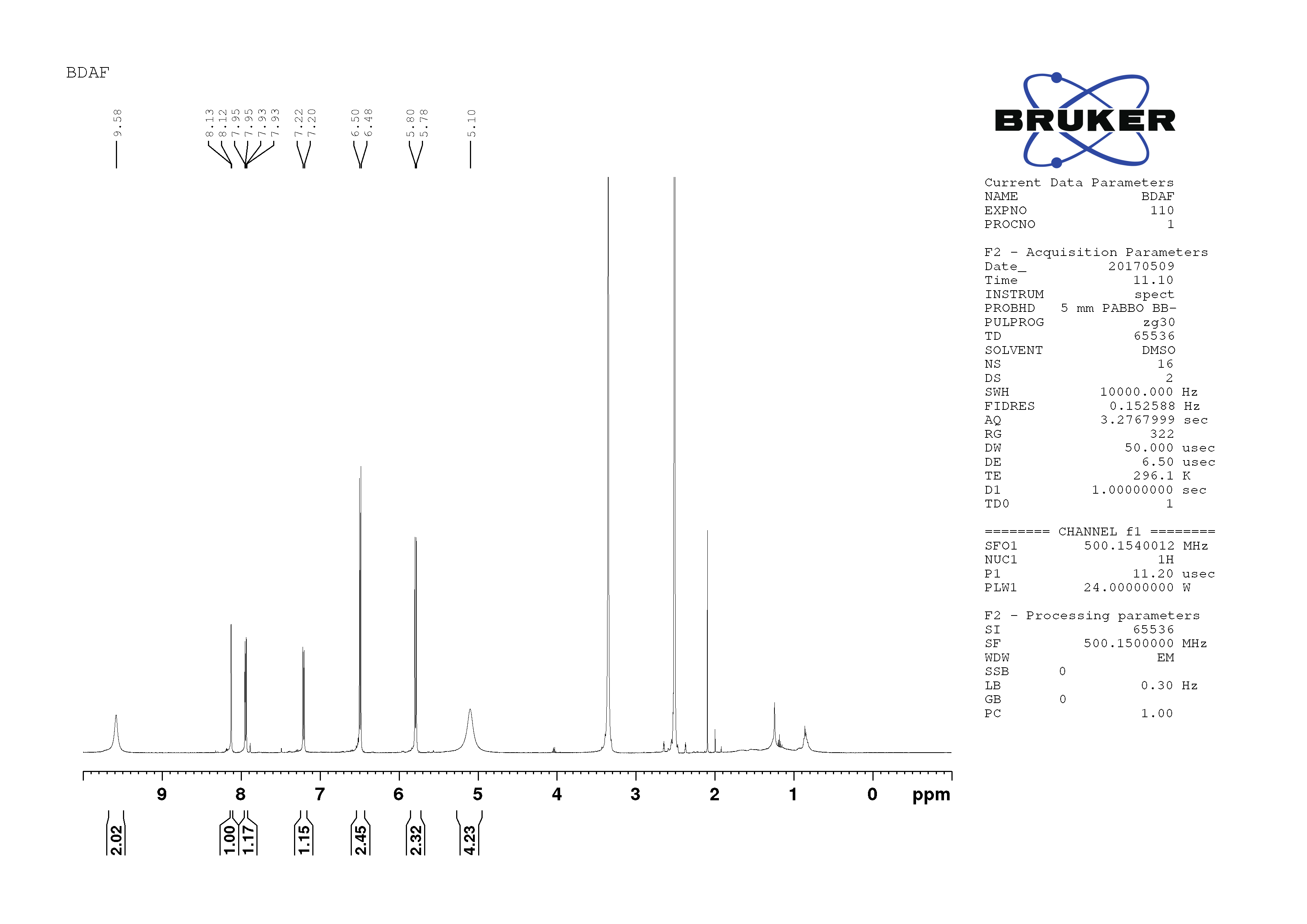


**Figure S11.** ^1^H NMR spectrum of **BDAF** in DMSO-*d*_6_.

**
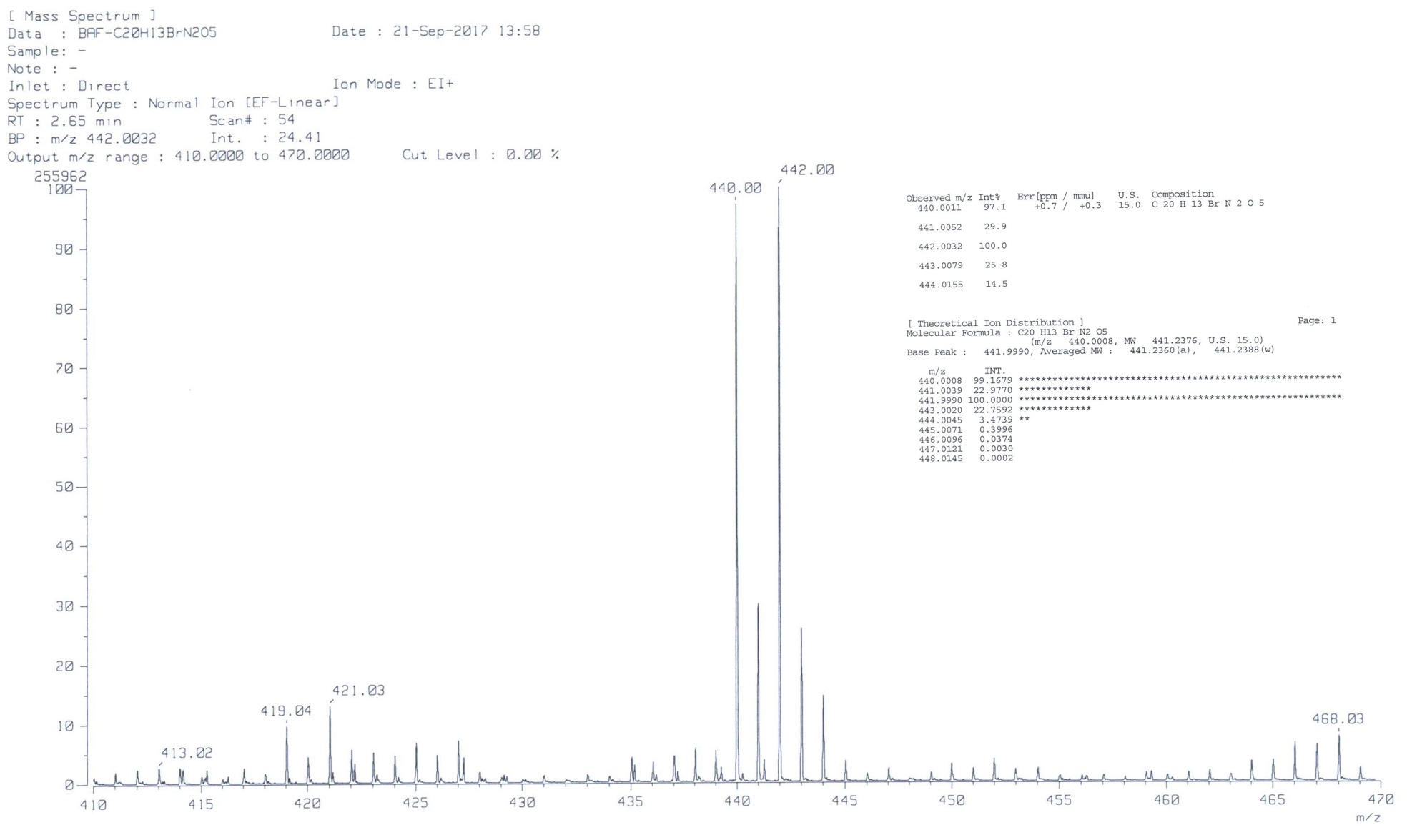
**

**Figure S12.** HRMS-EI spectrum of **BDAF**.


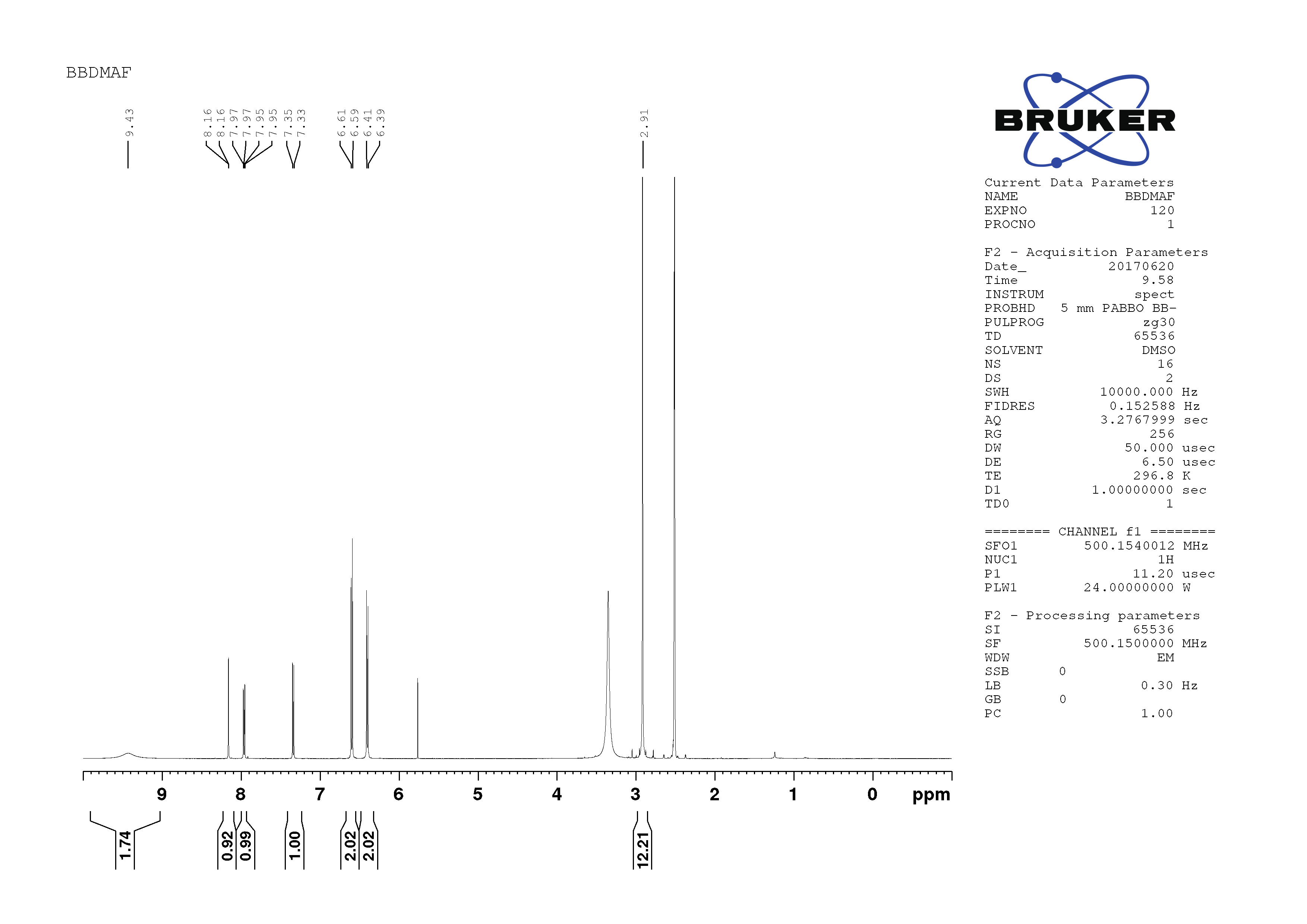


**Figure S13.** ^1^H NMR spectrum of **BBDMAF** in DMSO-*d*_6_.


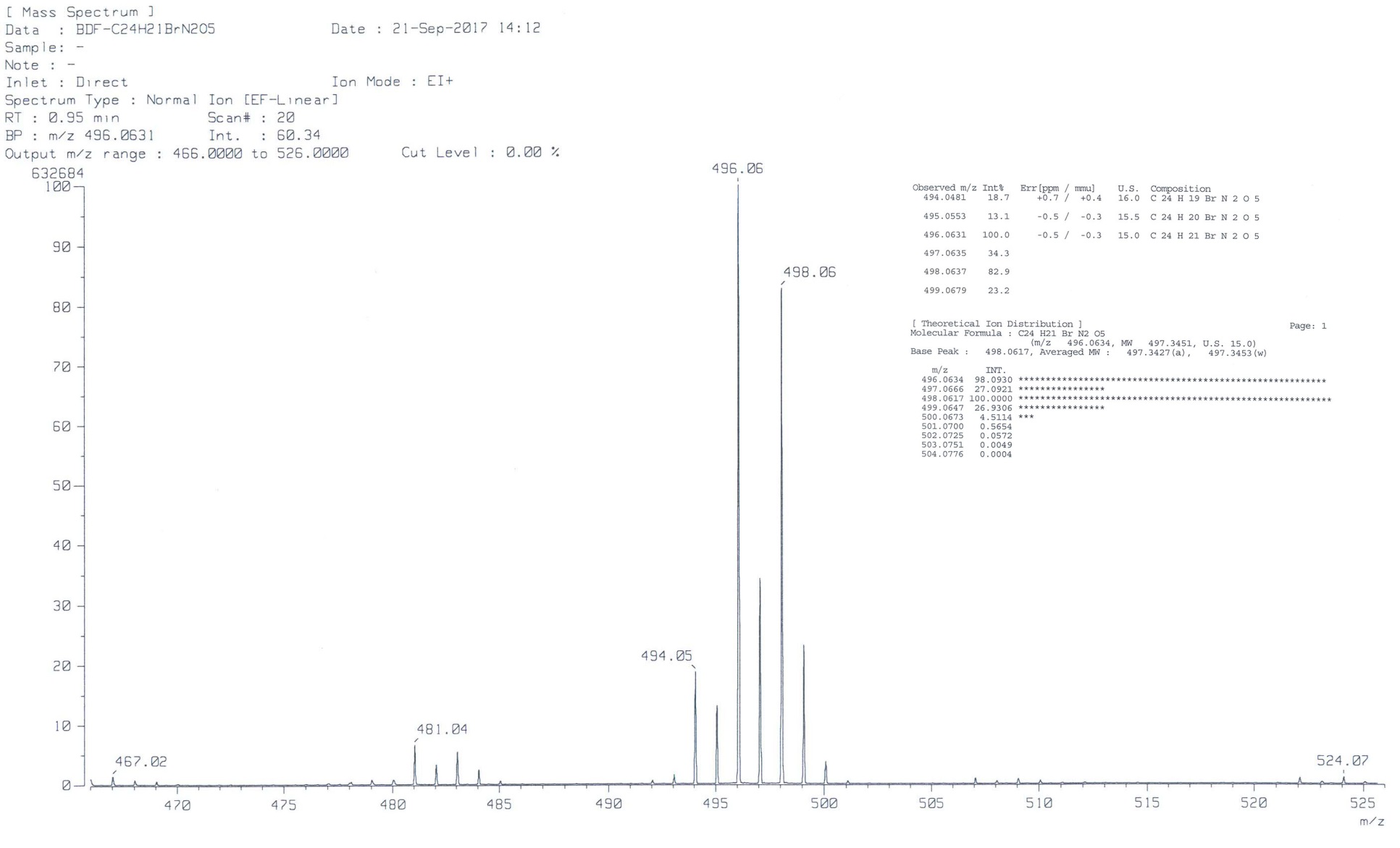


**Figure S14.** HRMS-EI spectrum of **BBDMAF**.


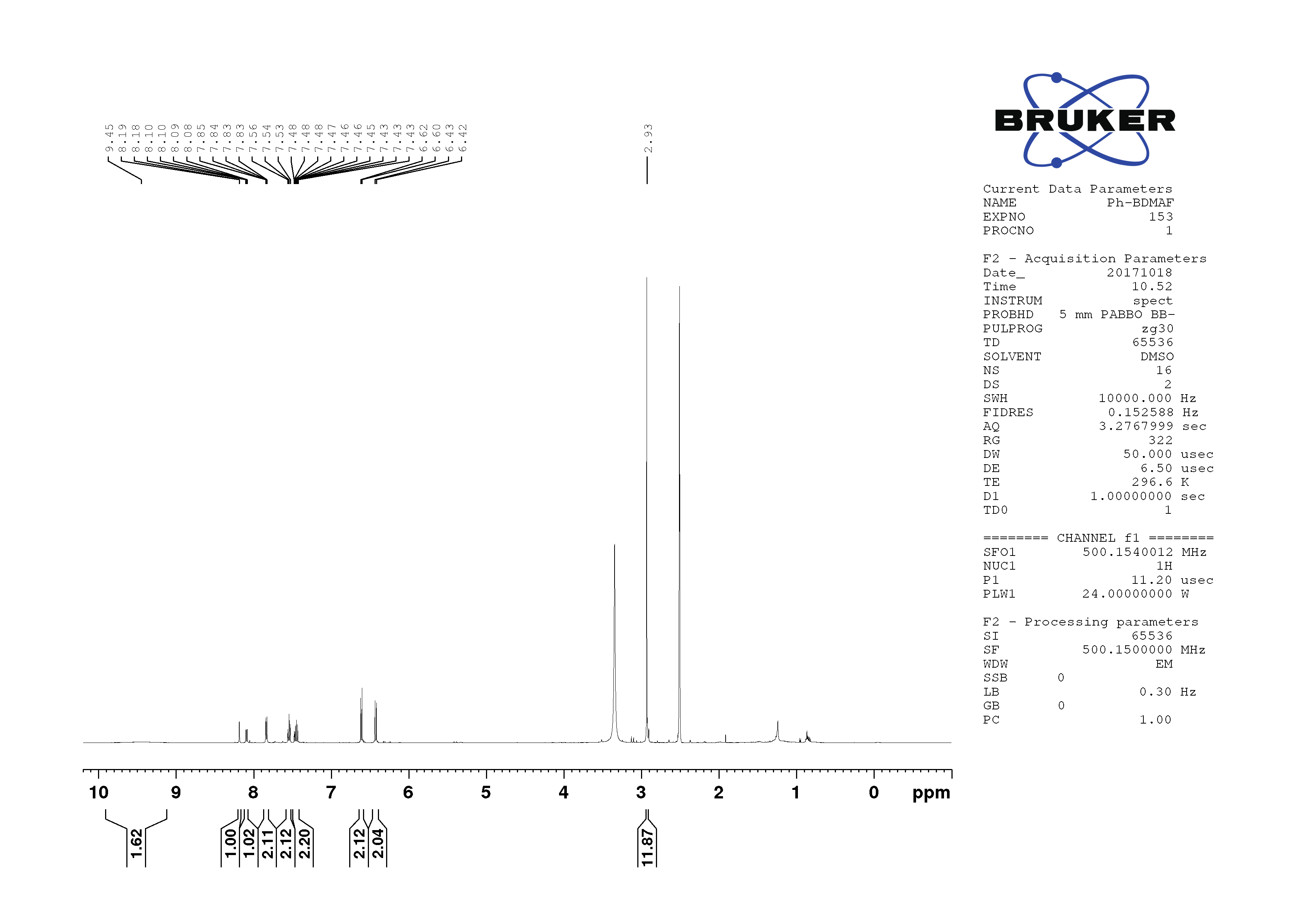


**Figure S15.** ^1^H NMR spectrum of **2** in DMSO-*d*_6_.


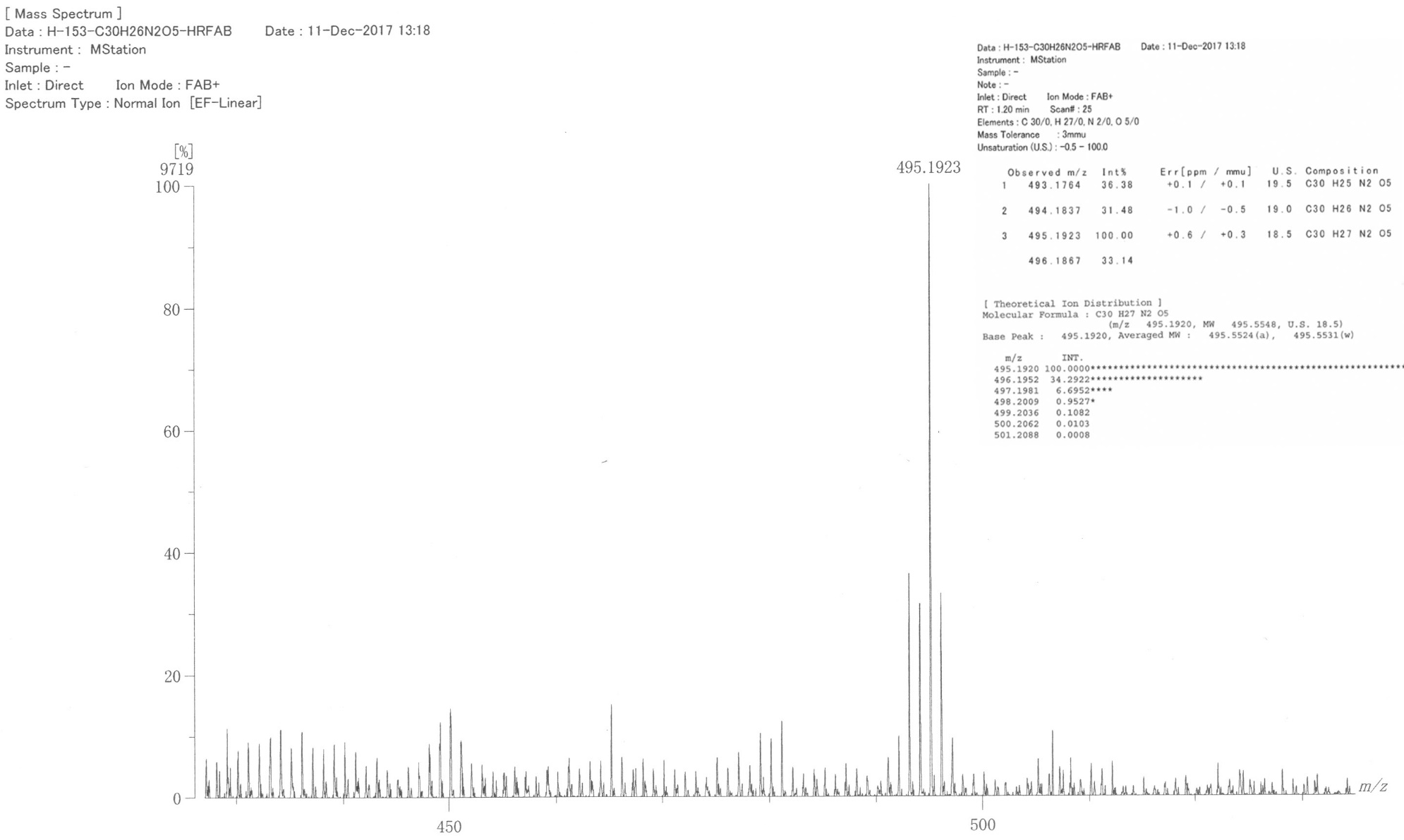


**Figure S16.** HRMS-FAB spectrum of **2**.

**
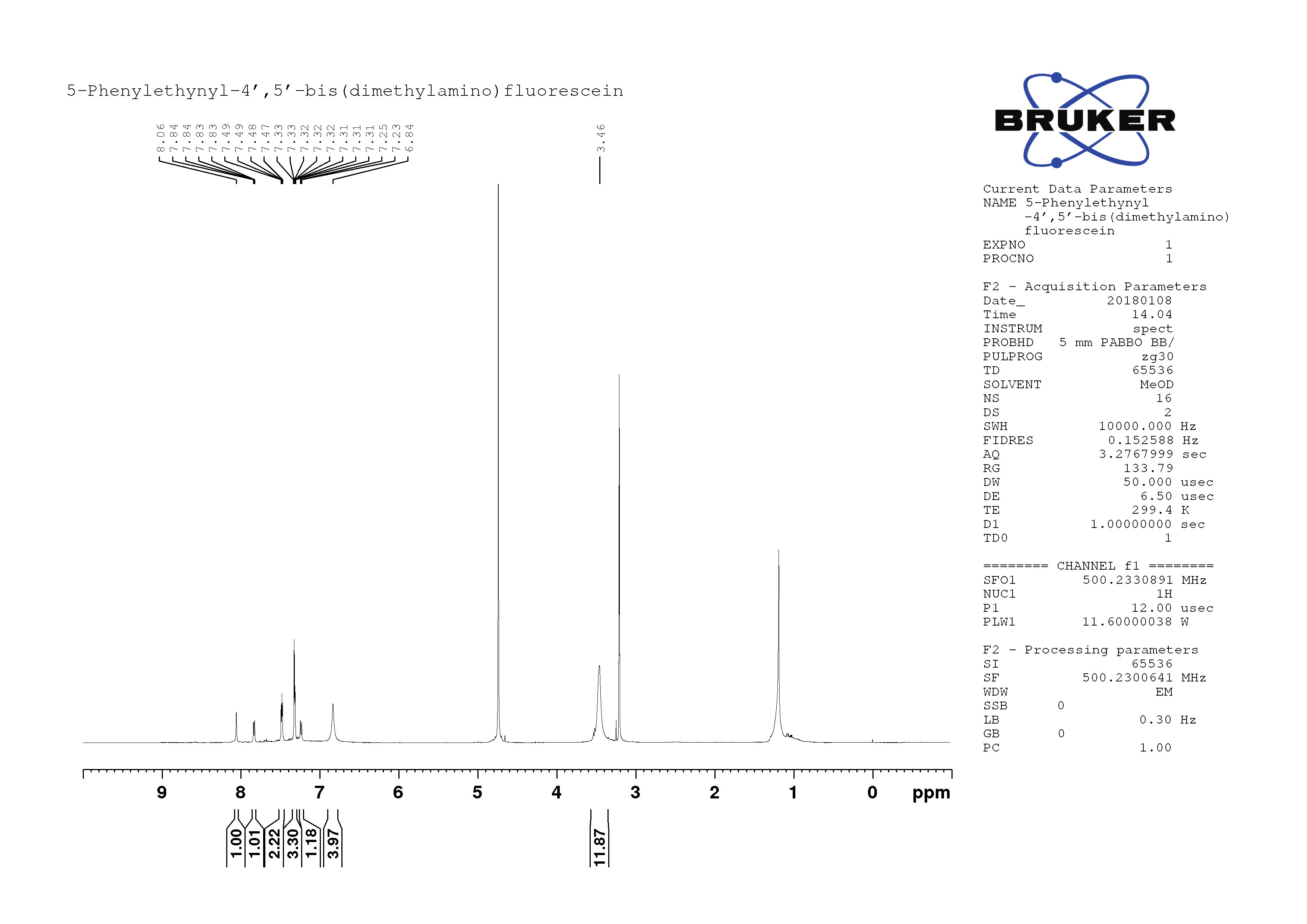
**

**Figure S17.** ^1^H NMR spectrum of **3** in DMSO-*d*_6_.

**
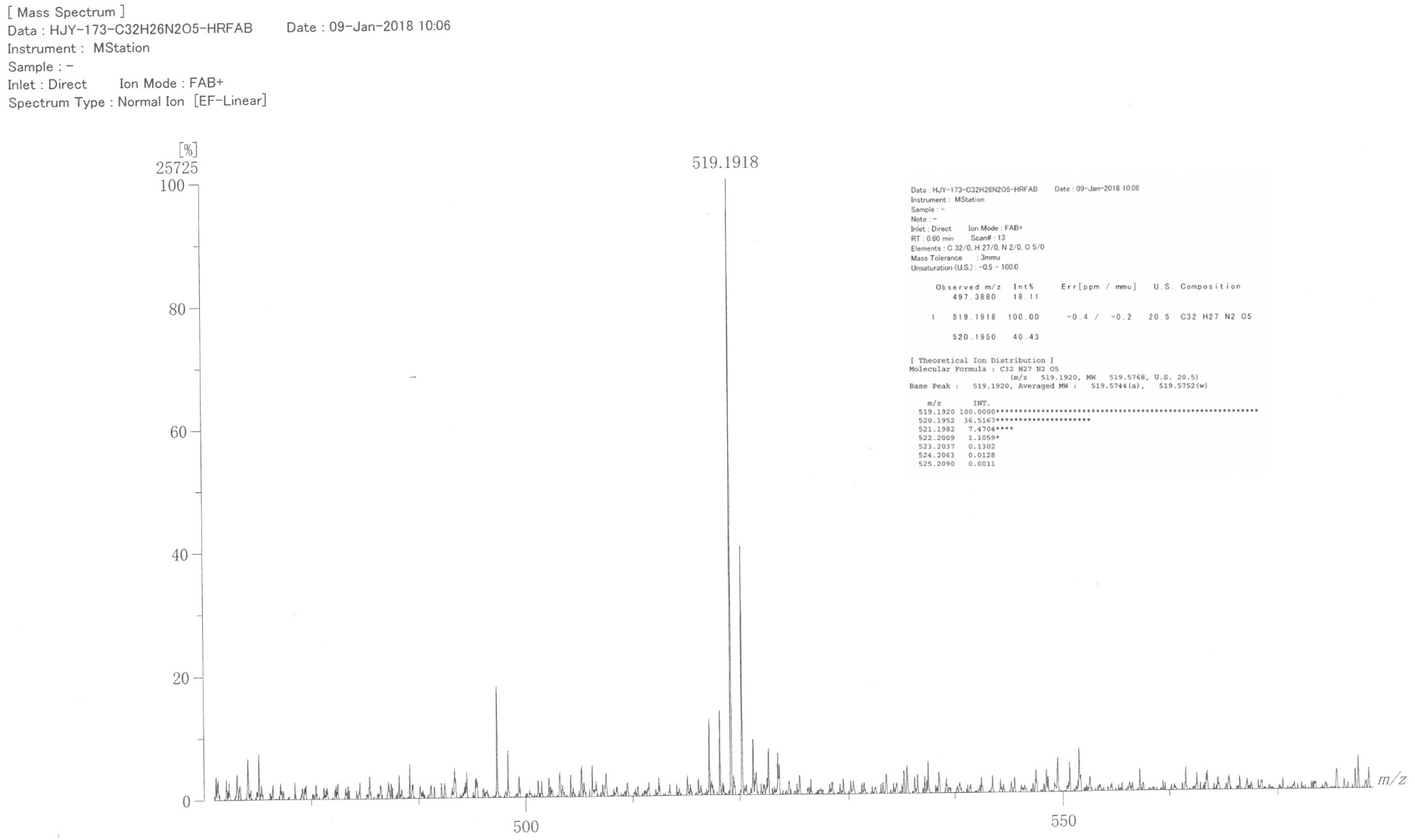
**

**Figure S18.** HRMS-FAB spectrum of **3**.
